# Th17 Cells Are Involved in the Local Control of Tumor Progression in Primary Intraocular Lymphoma

**DOI:** 10.1371/journal.pone.0024622

**Published:** 2011-09-20

**Authors:** Claire Galand, Sabrina Donnou, Lucile Crozet, Séverine Brunet, Valérie Touitou, Hanane Ouakrim, Wolf Herman Fridman, Catherine Sautès-Fridman, Sylvain Fisson

**Affiliations:** 1 Institut National de la Santé et de la Recherche Médicale, UMRS872, Centre de Recherche des Cordeliers, Paris, France; 2 Université Pierre et Marie Curie-Paris 6, UMRS 872, Paris, France; 3 Université Paris Descartes, UMRS 872, Paris, France; University Paris Sud, France

## Abstract

**Background:**

Th17 cells play an important role in the pathogenesis of many autoimmune diseases, but despite some reports of their antitumor properties, too little is known about their presence and role in cancers. Specifically, knowledge is sparse about the relation of Th17 to lymphoma microenvironments and, more particularly, to the microenvironment of primary intraocular B-cell lymphoma (PIOL), an aggressive lymphoma with a poor prognosis.

**Methods and Principal Findings:**

In this work, we investigated the presence of Th17 cells and their related cytokines in a syngeneic model of PIOL, a subtype of non-Hodgkin lymphoma. The very small number of lymphocytes trafficking in normal eyes, which represent a low background as compared to tumor-bearing eyes, allows us to develop the present model to characterize the different lymphocyte subsets present when a tumor is developing. IL-21 mRNA was expressed concomitantly with IL-17 mRNA in tumor-bearing eyes and intracellular expression of IL-17A and IL-21 in infiltrating CD4^+^ T lymphocytes. Interestingly, IL-17A production by T cells was negatively correlated with tumor burden. We also showed that IL-21 but not IL-17 inhibits tumor cell proliferation in vitro.

**Conclusions:**

These data demonstrate that IL-17A and IL-21-producing CD4^+^ T cells, referred as Th17 cells, infiltrate this tumor locally and suggest that Th17-related cytokines may counteract tumor progression via IL-21 production. Thus, Th17 cells or their related cytokines could be considered to be a new therapeutic approach for non-Hodgkin B-cell lymphomas, particularly those with an ocular localization.

## Introduction

Non-Hodgkin's lymphoma (NHL) is the major subtype of lymphoma and accounts for 3.4% of all cancer deaths [1]. Although it generally occurs in secondary lymphoid structures, extranodal growth can be observed, as in primary intraocular B-cell lymphoma (PIOL), an aggressive disease with a five-year survival rate after diagnosis of only 5% [2]. Too little is known about the role of the immune system in the progression of this disease. Our team developed a murine model to study its pathophysiological mechanisms [3]. Only few errant lymphocytes penetrate the eye in normal circumstances, it represents a small number of T cells. This means that the present model makes it possible to monitor tumor-infiltrating lymphocytes (TIL), especially useful because the presence of immune cells in the B-cell lymphoma microenvironment has been reported to be a good prognostic indicator for patient survival [4]. Activated CD4^+^ T cells might be a major participant in antitumor activity in this cancer [5]. Moreover increasing evidence suggests that infiltration of IL-17-producing CD4^+^ T cells regulates tumor progression [6]–[8]. These lymphocytes, called Th17 cells, usually produce IL-17, IL-21, and/or IL-22 [9], and help to clear pathogens [10]. They can also invade the eye in autoimmune diseases, such as autoimmune uveitis [11], [12]. Here we show that Th17 cells can be detected locally in the PIOL microenvironment. Additionally, IL-17 production is negatively associated with tumor burden, increasing as tumor burden decreases, and vice versa. Only IL-21, however, and not IL-17, has a direct antiproliferative effect on the tumor. Thus, modulation of Th17 cells or administration of IL-21 alone could be considered as a new therapeutic approach for non-Hodgkin B-cell lymphomas, particularly those with an ocular localization.

## Materials and Methods

### Mice

Six to 8-week-old female BALB/c ByJ mice (H2^d^) were purchased from Charles River Laboratories. Mice were provided with sterile food and water *ad libitum* and kept on a 12-hour light-dark cycle.

### Cells

A20.IIA (also called IIA1.6) is an FcγR-negative clone from the A20-2J murine lymphoma B-cell line [13]. A20.IIA cells were transfected with the GFP gene as previously described [3] and are hereafter referred to as A20.IIA-GFP cells. They were maintained at 37°C, 5% CO_2_ in complete Roswell Park Memorial Institute 1640 medium Glutamax plus (RPMI; Gibco-Invitrogen, France) containing 10% fetal calf serum (FCS; PAA laboratories, Germany), 100 U/ml penicillin and 100 µg/ml streptomycin (both from Eurobio, France), 10 mM sodium pyruvate (Gibco-Invitrogen), 50 µM 2-mercaptoethanol (Gibco-Invitrogen), and 0.5 mg/ml neomycin (G418; Gibco-Invitrogen). VAL is a human cell line derived from a diffuse large B-cell lymphoma (DLBCL) belonging to the NHL subtype and was cultured in the same conditions as A20.IIA-GFP, but without neomycin.

### Tumor implantation

Mice were first anesthetized by an intraperitoneal injection of 3.2 mg of ketamine (Virbac, France) and 0.16 mg of xylazine (Rompun 2%; Bayer Healthcare). A20.IIA-GFP (1.10^4^) cells in 2 µL phosphate buffer saline 1× pH 7.4 (PBS) were injected in aseptic conditions into the posterior chamber of the right eye with a 32-gauge needle attached to a syringe (Hamilton, Bonaduz AG). The same procedure was followed for control mice injected with PBS. Lacrinorm 2% (Bauch&Lomb) drops were instilled after injection. All animal studies were performed 19 days after tumor inoculation, conformed with European Union guidelines, and were approved by the Charles Darwin Ethics Committee in Animal Experiment, Paris, France (Permit Number: p3/2009/004).

### PCR

RNA from frozen enucleated eyes and from A20.IIA-GFP cells was extracted with an RNeasy Mini kit (Qiagen) in accordance with the supplier's instructions. The concentration in each sample was evaluated with a 2100 Bioanalyzer (Agilent Technologies), and total RNA was reverse-transcribed with the High Capacity cDNA Reverse Transcription kit (Applied Biosystems), in accordance with the manufacturer's instructions. Sequences for primers (Eurofins MWG operon, Germany) were as follows: HPRT forward: GGCCACAGGACTAGAACACC, HPRT reverse: ACAGGCCAGACTTTGTTGGA; GFP forward: ATGAAGATCGAGTGCCGC, GFP reverse: CACCACGAAGCTGTAGTA; IL-17A forward: TCCAGAAGGCCCTCAGACTA, IL-17A reverse: AGCATCTTCTCGAC-CCTGAA; IL-21 forward: GGGAATCTTCTCGGATCCTC, IL-21 reverse: AGGAGGGGAGGAAAGAAACA; IL-22 forward: GTCAACCGCACCTTTATGCT, IL-22 reverse: CATGTAGGGCTGGAACCTGT; IL-17RA forward: TGCCTGTGGCACTGAAGTAG, IL-17RA reverse: TTCATGGCTGCAGTGAAAAG; and IL-21R forward: ATGCGCTTGTCTCAATTCCT, IL-21R reverse: CACGTAGTTGGAGGGTTCGT. Primers were designed with the Primer3 website to amplify the cDNA fragments, that represent mature mRNA transcripts, of 220 bp for HPRT, 495 bp for GFP, 239 bp for IL-17A, 179 bp for IL-21, 189 bp for IL-22, 243 bp for IL-17RA, and 201 bp for IL-21R. Amplifications were conducted for 35 cycles on 40 (HPRT, GFP), 100 (IL-17R, IL-21R) or 120 ng cDNA. PCR products were separated and visualized on 1% agarose gel stained with ethidium bromide. The relative band intensity was determined with ImageJ software and the GFP, IL-17A, IL-21, and IL-22 bands were normalized to HPRT.

### Flow cytometry

Eyes were dissected in RPMI medium, digested with 0.1 mg/ml DNAse I (Roche Diagnostics, Meylan, France), and 1.67 Wûnch U/ml Liberase (Roche) at 37°C for 30 minutes, filtered and rinsed in PBS with 2 mM EDTA and 2% FCS. Cells were pre-incubated with 2.4G2 mAb (7 µg/ml) to block nonspecific binding to Fc receptors and then stained with the following mAbs: allophycocyanin-alexa fluor750 (APC-AF750)-labeled anti-CD3 (17A2, eBioscience), phycoerythrin-Texas red (PE-TR)-labeled anti-CD4 (RM4-5, Caltag), PE-labeled anti-CD19 (eBio1D3, eBioscience), AF647-labeled anti-IFNg (XMG1.2, BD Pharmingen), AF700-labeled anti-IL-17A (TC11-18H10.1, Biolegend), biotinylated anti-IL-21 (polyclonal, R&D), or AF647-labeled rat IgG1 (BD Pharmingen), and AF700-labeled rat IgG1(Biolegend) isotypic control mAb. The biotinylated mAbs were revealed by PE-cyanine5.5 streptavidin (eBioscience). For intracellular cytokine staining, cells were restimulated with phorbol myristate acetate (25 ng/ml, Sigma) and ionomycin (5 µg/ml, Alexis Biochemicals) for 4 hours in the presence of monensin (6.7 µM, Sigma) for the last 2 hours. After cell surface staining, intracellular staining was performed with the eBioscience kit and protocol (Foxp3 Staining Buffer Set). Cells were acquired on an LSR II cytometer and analyzed with Diva software (BD Biosciences).

### Cell cycle analysis

A20.IIA-GFP cells were cultured in the presence of 500 ng/ml murine IL-21. After 72 hours, cells were harvested, washed in cold PBS, and fixed with 2 ml of 70% cold ethanol in PBS for 2 hours at 4°C. After centrifugation, the supernatant was removed, and the pellet was rinsed with 0.5% PBS Tween and incubated with 50 µg RNAse A (Sigma-Aldrich) plus 50 µg propidium iodide (BD Bioscience) in PBS. Propidium iodide staining was determined with the LSR II cytometer.

### Cytokine release assay

Ocular cells (10^5^) were stimulated by beads coated with anti-CD3/CD28 monoclonal antibodies (Dynabeads, Dynal Biotech, Compiègne, France), as recommended by the manufacturer. To detect IL-17, the supernatants collected after 36 h were subjected to cytometric bead array analysis (CBA) in accordance with the manufacturer's instructions (functional beads, BD Biosciences; TC11-8H4 mAb anti-IL-17, Beckman Coulter; TC11-18H10 mAb PE-labeled anti-IL-17).

### Proliferation assay

A20.IIA-GFP cells (10^4^) were cultured in complete RPMI medium in 96-well plates in the presence or absence of a range of IL-17A (R&D Systems) or IL-21 (R&D Systems) concentrations (500, 100, 10, 1, 0.1 ng/ml) for 24, 48, or 72 hours. [^3^H]Thymidine (GE Healthcare) was added for the last 4 h of culture, and thymidine incorporation was assessed with a β-plate scintillation counter (Microbeta, Perkin Elmer). We used a purified anti-IL-21 antibody (Goat IgG, R&D) to neutralize murine 1L-21.

### Statistical analysis

Comparisons were tested with Student's t-test and the Mann-Whitney test, with GraphPad Prism (GraphPad Software, La Jolla, CA, USA). P values<.05 were considered significant.

## Results

### Th17 cells and related cytokines were present in the tumor microenvironment

**Figure 1:**
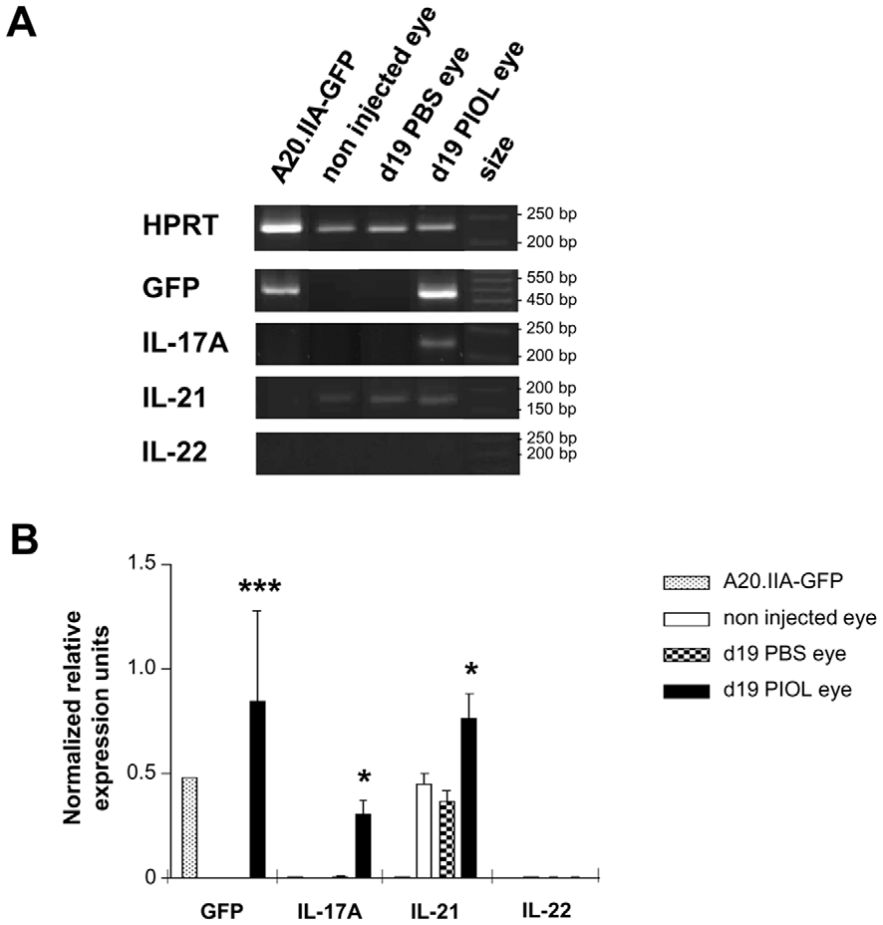
Th17-related cytokine transcripts in PIOL. (**A, B**) A20.IIA-GFP cells (1×10^4^) were inoculated into BALB/c mice on day 0. The mice were sacrificed on day 19. Analysis of HPRT, GFP, IL-17A, IL-21 and IL-22 mRNA expression by RT-PCR of uninjected eyes, PBS-injected eyes, PIOL eyes, and A20.IIA-GFP cells. (**A**) Representative gel migration from 4 different mice. (**B**) Densitometry analysis of the gel migration showing mean data from the 4 mice, representative of 2 different experiments. Each data is normalized to HPRT expression. Statistical analyses (Mann-Whitney tests) compare results from d19 PIOL eyes and d19 PBS eyes (*, p<.05). Error bars represent SEM.

A murine model of PIOL was generated by the injection of A20.IIA-GFP cells (H2^d^) into the posterior chamber of the eye of BALB/c mice (H2^d^). The presence of the Th17-related cytokine transcripts, IL-17A (IL-17), IL-21 and IL-22 mRNA, and of GFP was analyzed in extracts from the eyes 19 days after inoculation of tumor cells or injection of PBS. IL-21 but neither IL-17 nor IL-22 was expressed in both controls: uninjected and PBS-injected eyes (Fig. 1). By contrast, IL-17 and IL-22 could be detected in inflammatory positive control, lymph node derived from experimental autoimmune encephalomyelitis mouse (data not shown). In tumor-bearing eyes, IL-17 transcripts, but not IL-22 transcripts, were detected, and the expression of IL-21 was significantly higher than in the controls. Neither IL-17 nor IL-21 and IL-22 were detected in significant quantities in A20.IIA-GFP tumor cells (Fig. 1B).

**Figure 2:**
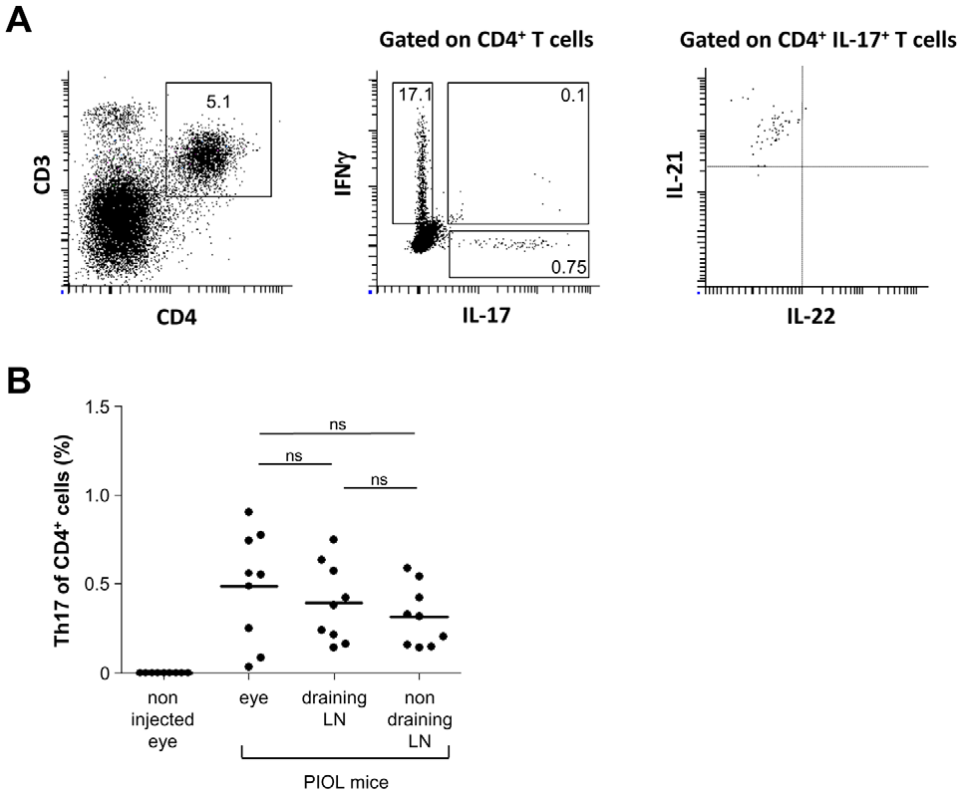
Th17 occurence in PIOL. (**A, B**) A20.IIA-GFP cells (1×10^4^) were inoculated into BALB/c mice at day 0; the mice were sacrificed on d19. (**A**) Representative Th17 cell staining (gated on CD4^+^IL-17^+^IL-21^+^ cells) analyzed by flow cytometry. (**B**) Th17 as a proportion of CD4^+^ T cells. The graph shows each data point from 10 mice. Data are representative of at least two independent experiments. Error bars represent SEM.

We previously showed that CD4^+^ T cells progressively infiltrate the eye after tumor inoculation. Intracellular cytometry showed the presence of Th17 cells, that is, CD4^+^ T cells coproducing IL-17 and IL-21, in eyes with lymphoma (Fig. 2A). IL-22 was not detected in these cells. Of note, a few double positive IFNγ^+^ plus IL-17^+^ cells were found (0.07±0.03% among CD4^+^ T cells). The mean frequency of Th17 cells among CD4^+^ T cells in PIOL eyes was 0.64±0.6%. This proportion is substantially lower than that of Th1 cells (defined as IFNγ^+^ CD4^+^ T cells) (17.2±6.8%). The uninjected eyes contained no Th17 cells. The proportion of Th17 cells in PIOL eyes was the same as in the lymph nodes of these mice, as shown in Fig. 2B. Taken together, these results demonstrate the presence of Th17 in the tumor microenvironment.

### High production of IL-17 was associated with a low tumor burden

**Figure 3:**
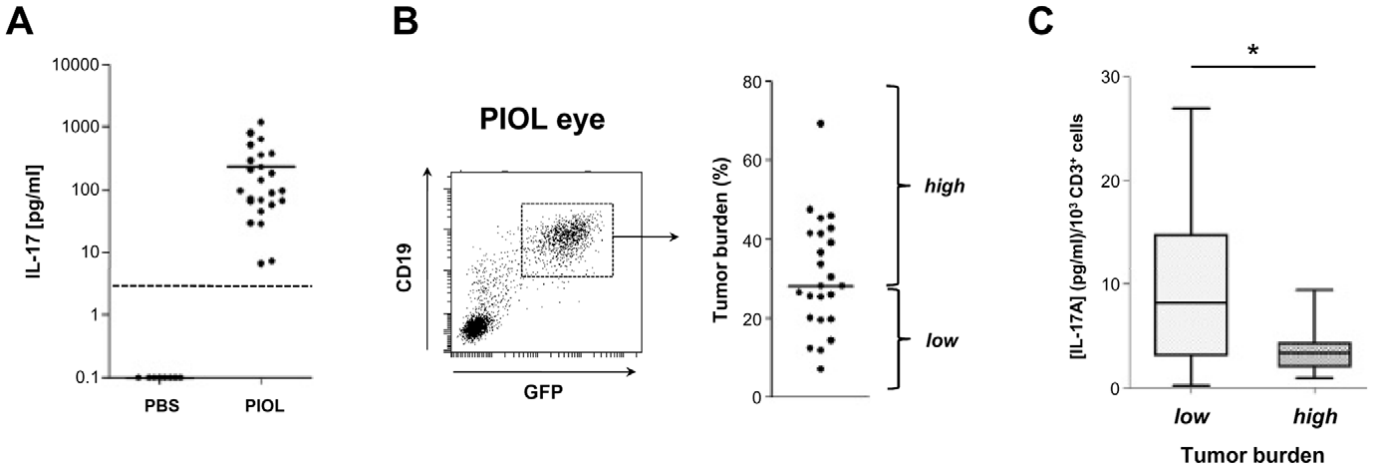
IL-17 secretion vs tumor progression in PIOL. (**A**) Ocular cells (10^4^; obtained from PBS- or A20.IIA-GFP-injected eyes) were stimulated in vitro with anti-CD3 and -CD28 microbeads. After 36 hours, culture supernatants were assayed for Il-17 secretion with a cytometric bead assay. Each data point corresponds to the result in an individual eye (n = 10) and the horizontal black bars symbolize the mean of the respective results. Dashed lines: baseline of detection for IL-17. Data are compiled from two independent experiments. (**B**) Left, representative tumor cell staining (gated on CD19^+^GFP^+^ cells) analyzed by flow cytometry; right, proportion of tumor cells among all live ocular cells. Low/high tumor burden cut-off is the median (horizontal bar) of a 24 mice group. (**C**) Correlation between tumor burden and in vitro IL-17 secretion of T cells from PIOL eyes. IL-17 secretion is normalized to the number of CD3^+^ cells for each eye. Comparison used the Student t test (*, p<.05).

To further analyze the role of Th17 cells in tumor growth, we investigated the level of ex vivo IL-17 secretion by TIL and its correlation with tumor burden (Fig. 3A). Supernatants of ocular cells were harvested from 24 eyes with lymphoma and stimulated by beads coated with anti-CD3 and anti-CD28 monoclonal antibodies: they contained from 6.7 to 1204 pg/ml of IL-17 (mean: 239 pg/ml). As expected, this assay, like the intracellular staining, found no IL-17 in either set of control eyes. Lymphomatous B cells were characterized by GFP and CD19 expression (Fig. 3B). Nineteen days after injection, tumor growth among the A20.IIA-GFP–injected mice was heterogeneous, with tumor cells accounting for a median of 28.4% of total ocular cells. Above and below this median, two groups were distinguished, one considered to have a high tumor burden, and the other a low burden. 1L-17 production differed significantly (*P* = 0.04) between these groups; it was low in the group with a high tumor burden, and vice versa (Fig. 3C). IWe therefore hypothesized that this cytokine has antitumor functions.

### IL-21 but not IL-17 had antitumor effects on lymphomatous B cells

**Figure 4:**
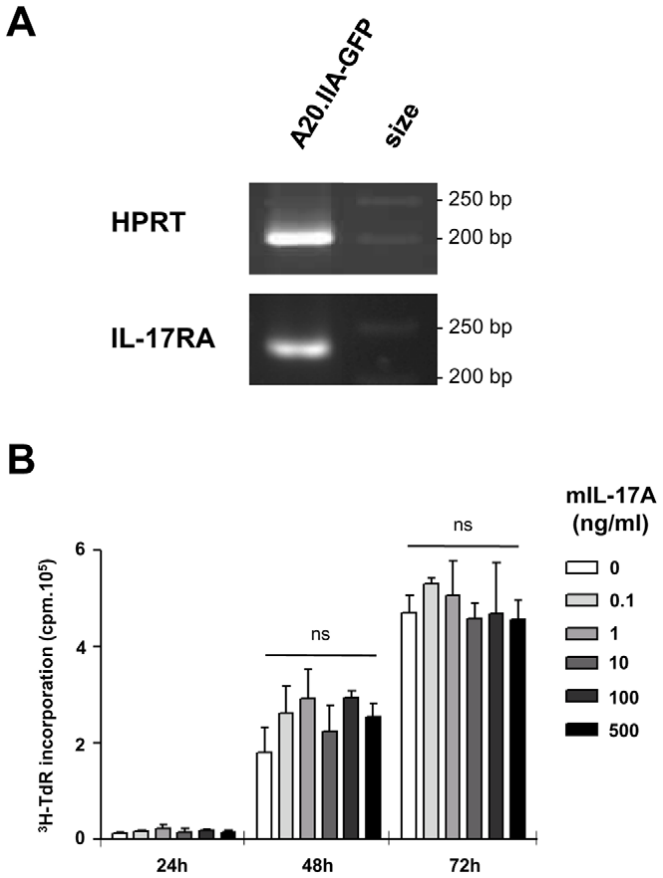
IL-17 has no effect on lymphoma B cells. (**A**) Analysis of IL-17RA mRNA expression by RT-PCR of A20.IIA-GFP cells. (**B**) Proliferation assay with a [^3^H]-thymidine incorporation to evaluate the effect of murine IL-17 (mIL-17) on A20.IIA-GFP cells. Data are representative of at least two independent experiments. Error bars represent SD.

**Figure 5:**
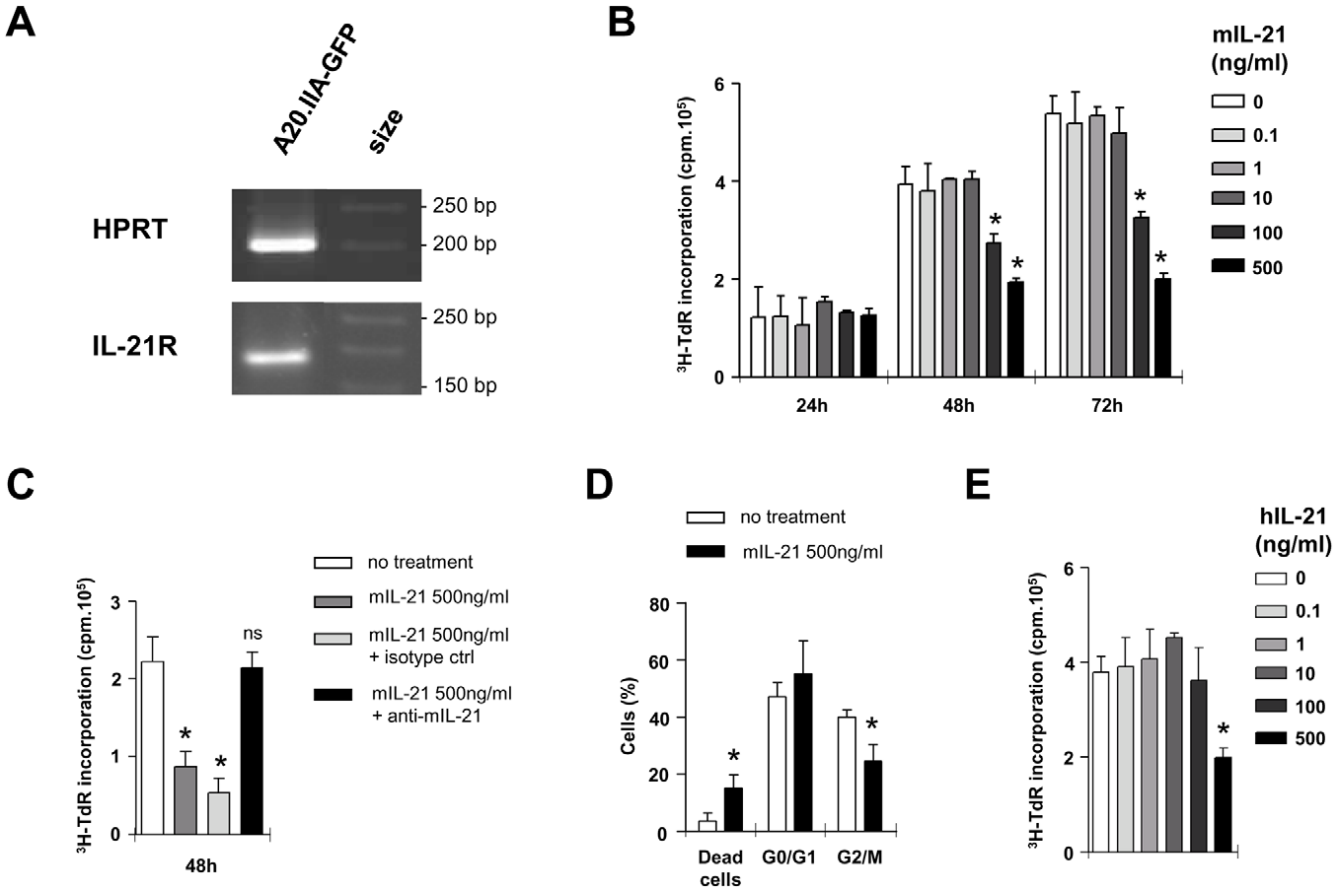
IL-21 effect on lymphomatous B-cells. (**A**) Analysis of IL-21R mRNA expression by RT-PCR of A20.IIA-GFP cells. (**B–D**) Proliferation assay with a [^3^H]-thymidine incorporation to evaluate effects of murine IL-21 (mIL-21) on A20.IIA-GFP cells. (**C**) Murine IL-21 effect is blocked by 30 µg/ml neutralizing anti-mIL-21 Ab. (**D**) Effect of mIL-21 on cell cycle of murine A20.IIA-GFP cells. Data are representative of at least two independent experiments. Error bars represent SD. (**E**) Human IL-21 (hIL-21) effect on VAL cell line proliferation. Comparison with untreated cells was tested with Mann-Whitney analysis (*, p<.05; **, p<.001).

To determine whether lymphomatous B cells were sensitive to IL-17, we tested for expression of the IL-17 receptor A chain and found it to be positive (Fig. 4A). We then measured the effect of increasing doses of mIL-17 on tritium-thymidine (^3^H-TdR) incorporation of A20.IIA-GFP cells (Fig. 4B). The presence of IL-17, even at high concentrations, did not change tumor proliferation at 72 h. IL-21, that was shown to be produced by Th17 cells in eyes with lymphoma (Fig. 2), has already been shown to act on B cells. We therefore investigated whether IL-21 affected A20.IIA-GFP cell proliferation. Results showed that IL-21R was expressed in the cell line (Fig. 5A) and that IL-21 induced a decrease in thymidine incorporation after 48 hours of incubation with at least 100 ng/ml of cytokine (Fig. 5B). The specificity of IL-21 activity on tumor cells was tested in vitro with a neutralizing antibody against this cytokine (Fig. 5C). As expected, background levels of proliferation were observed with cells incubated with 500 ng/ml of IL-21. Thymidine incorporation levels of A20.IIA-GFP cells rose to the levels of untreated cells on addition of 30 µg/ml of anti-IL-21 but not of the control isotype. Analysis of the IL-21-treated cells showed a significant increase in the percentage of dead cells (P = 0.02) and a significant decrease in the proportion of dividing cells — in either G2 phase or mitosis (P = 0.01) (Fig. 5D). We also tested whether the VAL lymphomatous B cells, from a human DLBCL-derived cell line, were sensitive to IL-21 in the same conditions (Fig. 5E). Incubation of these cells with 500 ng/ml of human IL-21 reduced their level of thymidine incorporation.

## Discussion

Th17 cells have previously been found in cancer microenvironments [14]–[17], but their functions remain unclear. On the one hand, adoptive transfer of in vitro polarized Th17 cells can promote cytotoxicity against tumor cells [7] and eradicate the tumor [8]. On the other hand, Th17 cells can increase neutrophil recruitment which in turn can exert protumorigenic activity [18], [19].

Our results demonstrated that in our lymphomatous microenvironment, Th17 cells had a standard phenotype (CD3^+^CD4^+^IL-17^+^IL-21^+^) [20], did not produce IFNγ, and did not express Foxp3 (data not shown). They accounted for 0.5% of the CD4^+^ T-lymphocyte population in the eye microenvironment and in draining and non draining lymph nodes. These data are consistent with results from Yang et al. [21], who analyzed human DLBCL, the NHL subtype of PIOL [22], and found a small proportion of Th17 cells, defined as CD4^+^IL-17^+^IFNγ^+^ or IFNγ^−^ T cells in the human lymphoma microenvironment. Interestingly, Th17 number is higher in the draining lymph node as compared to the non draining ones as well as the total mononuclear cell number (data not shown) suggesting that tumor triggers a localized immune response.

IL-17 and IL-21 have been described as Th17-related cytokines [23]. Their transcripts were found in tumor-bearing eyes on day 19. Interestingly, the PBS-injected eyes expressed IL-21 but not IL-17. IL-21 is most often reported to be produced by T lymphocytes [24]. Nevertheless more recently, its production has been shown in brain neurons [25]. It is therefore interesting to explore IL-21 expression by ocular cells and in particular retinal neurons.

Although it has been convincingly shown that endogenous IL-17 increases tumor burden in immunodeficient mice [26], it has the opposite effect in immunocompetent mice [27]. We expected as we developed the first syngeneic model of intraocular B-cell lymphoma in immunocompetent mice [28] that Th17 cells would have antitumor effects. Ex vivo secretion of this cytokine was negatively associated with tumor progression. Although the A20.IIA-GFP B cells expressed IL-17R, IL-17 had no direct antiproliferative effects. This result is consistent with the literature: other studies have failed to find any direct effect by IL-17 on other tumor growth [26], [29], [30]. In view of the significant negative correlation between IL-17 secretion and tumor burden, IL-17 might be only a reflection of Th17 activity. PIOL Th17 cells also produce IL-21, which has previously been reported to act on human lymphomatous B cells [31]. We showed here that IL-21 acts directly on the A20.IIA-GFP cell line inducing cell death and a decreasing of proliferation. The direct effect of IL-21 was confirmed on a human VAL DLBCL cell line in the same range of doses as for mice. IL-21 has already been tested in a clinical trial on NHL [32]. It would be interesting to test intraocular injections of IL-21 in PIOL.

Moreover, we hypothesize that Th17 cells act on tumors by secreting IL-21. However, no detectable IL-21 secretion was measured in the supernatant of total ocular cells from ex vivo cultures of tumor-bearing eyes (data not shown). IL-21 might be captured by cells that express IL-21R [33]— either tumor cells, as we demonstrated in this study, or Th17 cells themselves. If so, boosting Th17 cells may prove to be an effective therapeutic strategy in PIOL.
